# Working memory and intelligibility of hearing-aid processed speech

**DOI:** 10.3389/fpsyg.2015.00526

**Published:** 2015-05-07

**Authors:** Pamela E. Souza, Kathryn H. Arehart, Jing Shen, Melinda Anderson, James M. Kates

**Affiliations:** ^1^Department of Communication Sciences and Disorders, Northwestern UniversityEvanston, IL, USA; ^2^Knowles Hearing Center, Northwestern UniversityEvanston, IL, USA; ^3^Department of Speech, Language and Hearing Sciences, University of Colorado at BoulderBoulder, CO, USA

**Keywords:** aging, cognition, hearing loss, hearing aid, compression, quality, intelligibility

## Abstract

Previous work suggested that individuals with low *working memory capacity* may be at a disadvantage in adverse listening environments, including situations with background noise or substantial modification of the acoustic signal. This study explored the relationship between patient factors (including working memory capacity) and intelligibility and quality of modified speech for older individuals with sensorineural hearing loss. The modification was created using a combination of hearing aid processing [wide-dynamic range compression (WDRC) and frequency compression (FC)] applied to sentences in multitalker babble. The extent of signal modification was quantified via an envelope fidelity index. We also explored the contribution of components of working memory by including measures of processing speed and executive function. We hypothesized that listeners with low working memory capacity would perform more poorly than those with high working memory capacity across all situations, and would also be differentially affected by high amounts of signal modification. Results showed a significant effect of working memory capacity for speech intelligibility, and an interaction between working memory, amount of hearing loss and signal modification. Signal modification was the major predictor of quality ratings. These data add to the literature on hearing-aid processing and working memory by suggesting that the working memory-intelligibility effects may be related to aggregate signal fidelity, rather than to the specific signal manipulation. They also suggest that for individuals with low working memory capacity, sensorineural loss may be most appropriately addressed with WDRC and/or FC parameters that maintain the fidelity of the signal envelope.

## Introduction

Individuals with hearing loss must frequently communicate under adverse conditions, including noisy, reverberant, or otherwise distorted speech. The ability to communicate in adverse listening environments is reduced by hearing loss, or when the individual is older (e.g., Pichora-Fuller and Souza, 2003). More recently, it has been proposed that individuals with low *working memory capacity* may also be at a disadvantage in adverse listening environments. Working memory capacity refers to the ability to simultaneously process and store information (Baddeley, 1992). During speech perception, listeners must extract meaning from acoustic patterns and store that meaning for integration with the ongoing auditory stream. When acoustic patterns are degraded or altered from their expected form, it may be more difficult to match those acoustic patterns to stored lexical information (Rönnberg et al., 2013), and working memory may be engaged to a greater extent.

In the working memory model outlined by Baddeley (2000), the component of executive function (i.e., central executive) was included as the most important part of the working memory system. Its role was thought to be supervising, planning, and activating intentional actions. Other researchers' work illustrated this view more explicitly and defined executive function as shifting, updating, and inhibition in information processing (Miyake et al., 2000). In addition, speed of processing simple information was linked to working memory capacity in both older adults and children (Salthouse, 1991, 2000; Fry and Hale, 1996). These researchers proposed that individual difference in working memory capacity might be mediated by processing speed. Following from this idea, executive function and processing speed may also be related to signal modification in adverse listening conditions, consistent with the Ease of Language Understanding model (Rönnberg et al., 2013).

A common example of signal modification is speech in background noise. Everyday signal-to-noise ratios range from about +15 dB to as poor as −10 dB, with the most adverse situations including conversations in restaurants, automobiles, and public transportation (Olsen, 1998; Hodgson et al., 2007; Smeda et al., 2015). Listeners with low working memory capacity have more difficulty recognizing speech in noise than listeners with high working memory capacity (see Akeroyd, 2008 and Besser et al., 2013 for reviews). The association is stronger between verbal working memory tests and sentence intelligibility; and weaker between non-verbal working memory tests and syllables (e.g., Humes and Floyd, 2005). Moreover, some studies have shown a stronger relationship between working memory and sentence intelligibility when the sentences are presented at conversational or weaker levels to individuals with hearing loss (Humes and Floyd, 2005); or when the sentences are presented in modulated rather than unmodulated background noise (e.g., George et al., 2007). Presumably, both scenarios increase the number of inaudible or partially audible phonemes and the overall difficulty of the task, engaging working memory to a greater extent. The data on working memory capacity and speech in noise, then, are broadly consistent with the Rönnberg model.

While there are a large number of studies which measured working memory for speech in background noise, less information is available regarding other types of signal modification. For listeners with hearing loss, a potential source of signal modification is the signal processing applied by hearing aids. Only two decades ago, hearing aids were simple amplifiers where gain was dictated by the extent of hearing loss at each frequency, plus some means of limiting maximum output. Today, even “entry-level” hearing aids feature multiple features which may significantly modify the speech signal. Those features may include multichannel compression and output limiting, noise reduction, feedback suppression, and adaptive microphone directionality. Each feature has potential to alter the signal in a manner which may have consequences for the listener.

To illustrate this idea, consider wide-dynamic range compression (WDRC). WDRC is a core feature of digital hearing aids by which time-varying gain is applied to improve audibility of weak sounds while maintaining loudness comfort for higher-intensity sounds. The acoustic consequences of WDRC are dictated, in part, by the speed of the gain adjustment (i.e., attack and release times). In theory, fast compression which increases gain for brief speech segments will achieve greater consonant audibility than slow compression (e.g., Jenstad and Souza, 2005), and such compression is implemented in many commercial products. However, there is also evidence that alteration of the speech amplitude envelope—as will occur with fast compression (Kates, 2008)—may create a type of adverse listening situation for listeners who rely on envelope cues. A number of studies support the idea that listeners with low working memory capacity perform better with slow-acting than with fast-acting WDRC (e.g., Gatehouse et al., 2006; Lunner and Sundewall-Thoren, 2007; Davies-Venn and Souza, 2014; Ohlenforst et al., 2014; Souza and Sirow, 2014). Those data have been interpreted as a greater susceptibility to signal modification with low working memory capacity, which offsets the expected benefits of improved consonant audibility.

If susceptibility to signal modification is related to working memory capacity, we would expect to see similar patterns for other types of hearing-aid processing. One such example is frequency compression (FC). For listeners with substantial high-frequency loss, high-frequency gain may not result in audibility, either because gain is limited by the electroacoustic characteristics of the device, or because the listener may not have sufficient receptor cells to receive the amplified high-frequency cues (Moore, 2004). In FC, signal energy at high frequencies is digitally compressed into a lower frequency region where the listener has better hearing acuity. As with WDRC, the intent is to improve signal audibility. However, as with fast-acting WDRC, improved audibility requires signal modification. FC alters harmonic spacing and modifies spectral peak levels (McDermott, 2011). If the benefits of FC outweigh the (potential) disadvantage of such modification, speech intelligibility may be improved by signal modification (e.g., Souza et al., 2013; Alexander et al., 2014; Ellis and Munro, 2015). However, FC which results in extensive signal modification could also be viewed as creating an adverse listening environment for some listeners. Recent data show that the benefit of FC is influenced by working memory capacity, as well as age and amount of hearing loss (Arehart et al., 2013a; Kates et al., 2013). As with fast-acting WDRC, the FC data can be interpreted to show that listeners with low working memory capacity have greater susceptibility to signal modification caused by hearing-aid processing.

Although varying a single hearing-aid parameter is a reasonable way to model (potential) adverse listening situations for hearing-aid wearers, such implementations may not generalize to wearable hearing aids in which multiple parameters interact with (and perhaps offset) one another. We know that when signal processing algorithms are combined, speech intelligibility and quality ratings are different than when the algorithms process the same speech in isolation (e.g., Franck et al., 1999; Chung, 2007; Anderson et al., 2009). Related to working memory, recent work by Neher and colleagues (Neher et al., 2013, 2014; Neher, 2014) explored the relationship between working memory, executive function, and response to aggregate signal modification. In Neher's work, signal modification was created by a combination of background noise, hearing aid noise reduction and directional microphones. The extent of signal modification was manipulated by controlling the level of background noise and/or the strength of the noise reduction algorithm. Consistent with (Arehart et al., 2013b), more aggressive noise reduction was verified to result in greater signal modification. In agreement with previous work for other types of hearing aid processing, working memory capacity and amount of hearing loss predicted amplified speech intelligibility.

To summarize, a growing body of work suggests that a relationship between working memory capacity and listening in adverse conditions can be demonstrated not only for environmental distortions such as background noise (Akeroyd, 2008), but for signal modification introduced by hearing devices. In this study, we explored the relationship between signal modification, speech intelligibility, and working memory capacity, where signal modification was the aggregate effect of background noise and simulated amplification with two processing strategies: amplitude compression, and FC. Each strategy was further manipulated by applying parameters which would modify the signal to a greater or lesser extent. Here, we hypothesize that signal modification created by amplification is related to working memory capacity, such that the resulting modification is the key factor. If that holds true, it would be consistent with Rönnberg and colleagues' model of working memory (Rönnberg et al., 2013), in which greater modification of the expected acoustic signal places a greater demand on working memory capacity. Participants were older adults with mild-to-moderate hearing loss. Working memory capacity was quantified using a reading span test (RST). Executive function and processing speed were also measured in order to evaluate their relationship to intelligibility of speech. We posed three questions: (1) How does the performance of speech intelligibility (and quality) vary across adverse listening conditions? (2) What role do listener factors such as cognitive ability, amount of hearing loss, and age have in speech intelligibility (and quality) performance under such adverse listening conditions? (3) Is there a cognitive factor (specifically, working memory capacity, executive function, or processing speed) that improves prediction of intelligibility in adverse listening conditions?

## Materials and methods

### Participants

**Figure 1:**
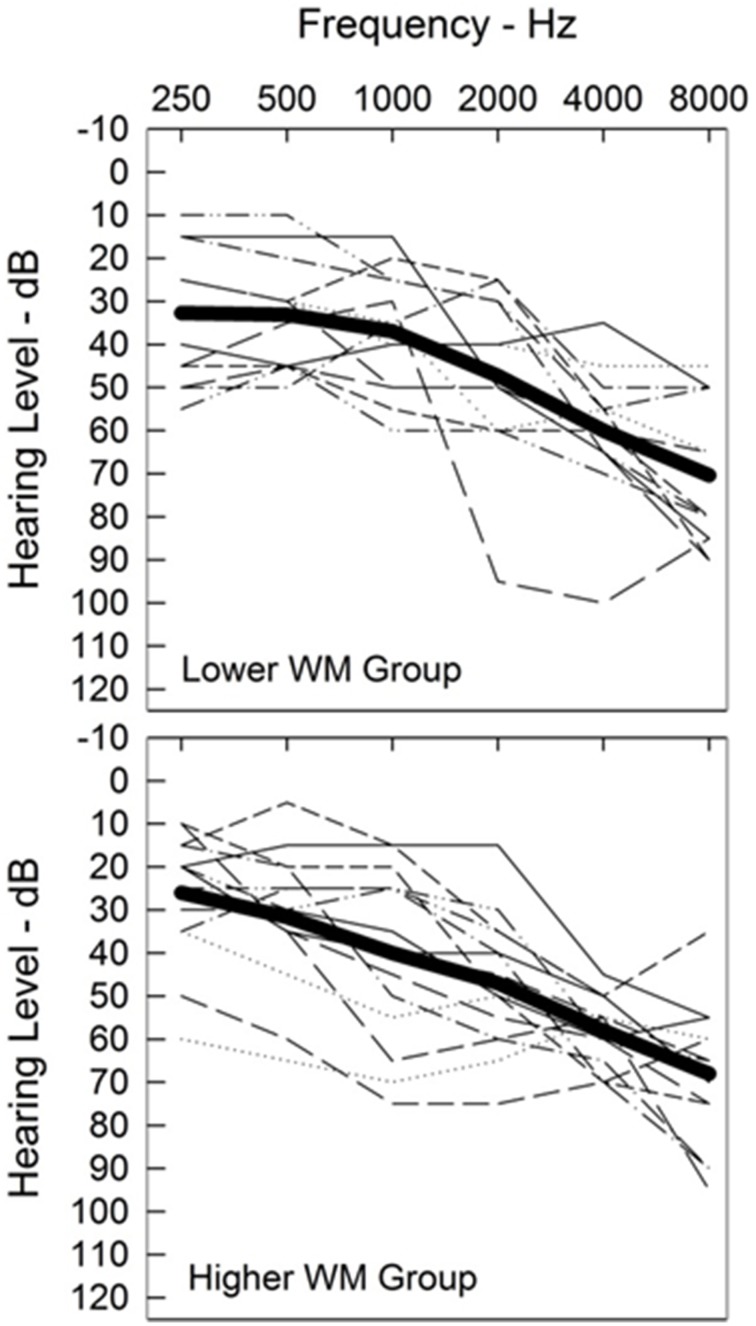
**Individual test-ear audiograms (thin lines)**. Audiograms are grouped by working memory capacity (described in detail later in this paper). Audiograms for participants with lower working memory capacity (WM) are shown in the top panel and with higher working memory in the bottom panel. The average audiogram for each group is shown with a heavy line.

Participants were recruited and data collected across two study sites (Northwestern University and University of Colorado), using identical test equipment and protocols. Twenty-nine older participants aged 49–89 years (mean age 74.0 years) participated in the study. Inclusion criteria included symmetrical sensorineural hearing loss with thresholds between 25 and 70 dB HL at octave frequencies between 0.5 and 3 kHz; a difference in pure-tone average [0.5, 1, 2 kHz] ≤ 10 dB across ears; and air-bone gaps ≤10 dB. One ear was randomly selected as the test ear for the auditory portions of the study. Test ear thresholds are shown in Figure 1, grouped by working memory capacity (explained in detail later in this paper). Quiet word recognition scores (monosyllabic words presented to the test ear at 30–40 dB SL) ranged from 68 to 100% (mean score 88%). All participants had good self-reported health, normal or corrected-to-normal vision, and completed a cognitive screening using the Montreal Cognitive Assessment (MoCA; Nasreddine et al., 2005). This brief (10 min) cognitive screening test assesses attention, working memory, executive function, visual-spatial ability, and language skills. Participants scoring 22 or higher on the MoCA were accepted into the study. That inclusion criterion considered the effects of hearing loss (Dupuis et al., 2013) and participant demographics (Rosetti et al., 2011), and was similar to that followed in previous studies with the same population (Anderson et al., 2012, 2013). Testing (audiometric evaluation, speech intelligibility, quality ratings, working memory capacity, executive function, and processing speed) was completed over test sessions of 1–2 h each, including test breaks. Ethical and safety review of the test protocol was conducted and approved by the local institutional review board at each site. Participants were compensated for their time.

### Working memory test

The RST (Daneman and Carpenter, 1980; Rönnberg et al., 1989) was used to measure working memory. The test was designed to measure individual working memory capacity in terms of coordinating storage and processing requirements simultaneously. During the test, 54 sentences were shown on the computer screen one word or word pair at a time (on-screen duration 800 ms). Half of the sentences were absurd (e.g., “The train” “sang” “a song”), and half were semantically meaningful (e.g. “The captain” “sailed” “his boat”). The participants were asked to read each sentence and make a semantic judgment as to the sense of the sentence. After each 3–6 sentence block, the participants were asked to recall the first or the last words of a presented set of sentences. The primary measure of the individual's working memory capacity was the proportion of words that were correctly recalled.

### Processing speed and executive function

The flanker task (Eriksen and Ericksen, 1974) was used to measure the participants' processing speed and executive function. In this task, the participants were asked to identify the direction of an arrow that was presented on the center of the screen. Processing speed was quantified by reaction time (in milliseconds) to a single arrow on the screen without any visual interference. Executive function was quantified by the difference in reaction time when the central arrow was flanked by arrows that had the same (congruent) vs. different (incongruent) directions as the center arrow.

The participants were seated in front of a computer monitor with eye-to-screen distance of 17 inches. They were asked to press the button corresponding to the direction of the arrow (i.e., press left button when the arrow pointed left, press right button when the arrow pointed right) as quickly and as accurately as possible. A practice block (8 trials for the processing speed test, 12 trials for the executive function test) was conducted prior to each test in order to ensure the instruction was followed. The processing speed test had one block of 40 trials. The arrow was pointing left in half of the trials and pointing right in the other half. The executive function test had one block of 80 trials. Three arrows on each side surrounded the center arrow in each trial. The side arrows were pointing to the same direction as the center arrow in half of the trials, while pointing a different direction in the other half. The order of the trials was randomized across participants.

### Speech intelligibility and quality stimuli

Speech intelligibility and quality were measured using materials drawn from the Institute of Electrical and Electronics Engineers sentence corpus (Rosenthal, 1969). This corpus consists of a large set of sentences which make semantic sense but contain relatively little contextual information. Each sentence includes five key words which can be scored for correct repetition (e.g., “The birch canoe slid on the smooth planks”; “Glue the sheet to the dark blue background.”). The sentences were spoken by a female talker and were digitized at a 44.1 kHz sampling rate and then downsampled to 22.05 kHz. The level of the sentences at the input to the hearing-aid simulation was set at 65 dB SPL. The final presentation level was based on the individualized frequency-gain shaping described below.

To create realistic adverse listening conditions, the sentences were digitally combined with multi-talker babble (Cox et al., 1987) at two signal-to-noise ratios, 0 and +10 dB, plus a quiet (no noise) condition. For each signal-to-noise ratio, the sentences were set to a level of 65 dB SPL and the noise level adjusted prior to mixing.

### Hearing aid processing

Dynamic-range compression (WDRC) was implemented using a hearing aid simulation program with 6-channel FIR filter bank. The center frequencies of the bands were 250, 500, 1000, 2000, 4000, and 6000 Hz. Inputs having intensities below a lower compression threshold (45 dB SPL) received linear amplification, and inputs above an upper compression threshold (100 dB SPL) received compression limiting to prevent over-amplification of intense sounds. Input levels between the two compression thresholds were subjected to WDRC with a compression ratio of 2:1. There were two WDRC conditions, with release times of 40 and 640 ms (re: ANSI, 2009). The attack time was set to 5 ms in both cases. In a control condition, linear processing was implemented using the same algorithm, but with the compression ratio set to 1:1.

FC was implemented using sinusoidal modeling (McAulay and Quatieri, 1986). The signal was separated into two frequency bands above and below each of the cutoff frequencies specified below. The low-frequency band was used without processing, while FC was applied to the high-frequency band using short-time frequency analysis, as follows: (1) the high-frequency signal was windowed in 6 ms segments using a von Hann raised-cosine window; (2) the shifted frequency components used the original amplitude and phase values, applied to sinusoids generated at the new frequencies; (3) the synthesized high-frequency and original low-frequency signals were recombined in the final step to produce the processed output.

Two FC conditions were used to present strong and mild signal modification (Strong: FC cutoff of 1000 Hz, FC ratio of 3:1; Mild: FC cutoff of 1500 Hz, FC ratio of 1.5:1). There was also a control condition with no FC applied to the signal.

To accommodate the individual hearing losses, all processed stimuli were amplified using the National Acoustics Laboratories-Revised (NAL-R) linear prescriptive formula (Byrne et al., 2001) with the gain implemented using a 128-point linear-phase FIR digital filter.

### Signal fidelity

Signal modifications to the original speech signal caused by cumulative effects of the additive noise and the signal processing were quantified using a signal fidelity metric (Kates and Arehart, 2014). The metric starts with an auditory model that reproduces the fundamental aspects of the auditory periphery including auditory frequency analysis, the dynamic-range compression mediated by the outer hair cells, firing-rate adaptation associated with the inner hair cells, and auditory threshold. The output of the auditory model is the speech envelope in 32 auditory frequency bands from 80 to 8000 Hz.

The envelope outputs from the model for an unmodified reference signal having no noise or distortion are compared to the model outputs for the degraded signal. At each time sample, a smoothed version of the auditory spectrum is formed. The variations as a function of time in the smoothed spectrum for the modified signal are compared to the variations in the reference signal using a normalized cross-correlation operation. The resultant metric thus combines (1) the accuracy in reproducing the short-time spectral shape across auditory bands and (2) the accuracy in reproducing the envelope temporal modulation within auditory bands. The metric therefore provides an overall measure of fidelity in reproducing the time-frequency modulation pattern of the modified signal in a manner consistent with the time-frequency modulation patterns of speech (Zahorian and Rothenberg, 1981). The metric values range from 0 to 1, with 0 indicating a complete lack of envelope fidelity relative to the reference and 1 indicating perfect envelope fidelity.

### Speech intelligibility

For the intelligibility tests, the participant was seated in a double-walled sound booth and listened to stimuli presented monaurally via a Sennheiser HD 25 1 II headphone in the better ear. Each trial consisted of a sentence randomly drawn from one of the 27 processing conditions (3 WDRC × 3 FC × 3 signal-to-noise ratios). Subjects first heard 27 practice sentences (1 from each test condition) and then listened to 270 test sentences (with 10 sentences in each condition). No feedback was provided. The timing of presentation was controlled by the participant. The participant repeated the sentence and scoring was completed by the experimenter, seated outside the sound booth. The order of sentences and conditions was randomized across listeners. Scores were calculated based on the proportion of correctly-identified key words (10 sentences per condition and 5 words per sentence for 50 key words per condition, per participant).

### Speech quality

In the speech quality task, listeners rated the sound quality of speech that had been modified according to processing conditions discussed above. Stimuli were spoken by a woman, and were two sentences taken form the IEEE corpus (“Take the winding path to reach the lake. A saw is a tool used for making boards”). Each trial included the same two sentences to limit the effects of intelligibility. Speech processed by hearing aid signal processing algorithms have been shown to be well predicted by metrics using a single “overall quality” rating scale (e.g., Arehart et al., 2010), even though sound quality is multidimensional in nature (Gabrielsson et al., 1988; Arehart et al., 2007). In this study, listeners used a computer-based slider bar to rate the sound quality using a rating scale from 0 (poor sound quality) to 10 (excellent sound quality) in 0.5 increment (ITU, 2003^1^). The participant controlled the timing of presentation. Testing was completed in four blocks. The first block was a practice block, and included one trial from each of the processing conditions. The practice block familiarized the listener with the task and process of using the rating scale. Three test blocks followed, with 45 trials per block. Processing conditions were presented five times each, and were randomized to occur at any point within the three test blocks. No feedback was provided.

## Results

### Working memory

**Figure 2:**
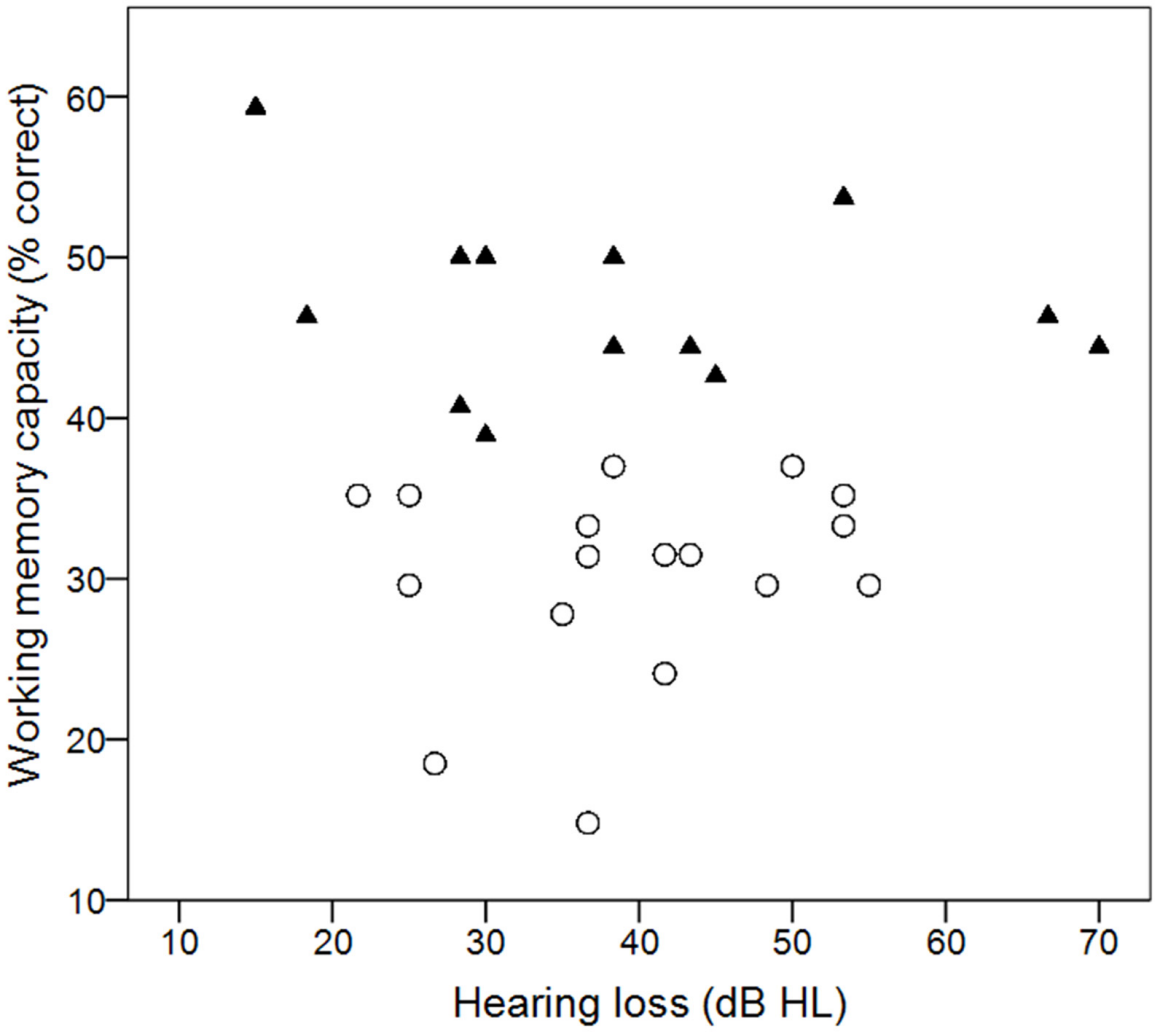
**Individual working memory scores as a function of hearing loss**. Filled triangles and open circles show individual scores that fall above or below the median score.

Individual working memory scores are plotted in Figure 2 as a function of amount of hearing loss (pure-tone average for 0.5, 1, 2 kHz). Scores ranged from 15 to 54%, with a mean score of 38%. The distribution of scores was similar to scores in previous studies which used the same reading span implementation, and where mean reading span scores ranged from 34 to 44% (e.g., Foo et al., 2007; Arehart et al., 2013a,b; Souza and Sirow, 2014). Within our test cohort there was no relationship between working memory capacity and amount of hearing loss (*r* = −0.045, *p* = 0.817). For some of the planned analyses, the participants were assigned to either a high (*n* = 13) or low (*n* = 16) working memory group, based on the median score for the group. Individuals who fell on the median were assigned to the higher group. Those groupings are indicated by different symbols in Figure 2.

### Statistical analysis

Similar to other work from our group (e.g., Arehart et al., 2013a), the primary analytical approach was hierarchical linear modeling (HLM) also known as multi-level modeling (Singer and Willett, 2003). Multi-level models were developed for the analysis of nested data structures or repeated measures data. They incorporate between-listener characteristics in models of individual performance across multiple conditions (Raudenbush and Bryk, 2002), so are well suited for research questions where the variability in outcomes may be a result of differences between groups as well as individual listener differences.

The analysis was conducted using HLM 6 (Raudenbush and Bryk, 2002) and included three different multi-level models. Each model considered signal modification (using the envelope fidelity metric described above), amount of hearing loss (expressed as the average of thresholds at 1, 2, 3, and 4 kHz in the test ear) and age; plus one of the cognitive measures (working memory capacity, executive function, or processing speed). Listeners were grouped for amount of hearing loss, working memory capacity, executive function, and processing speed using the median as the cutoff criteria. Individuals who fell on the median were assigned to the higher scoring group.

### Speech intelligibility

**Figure 3:**
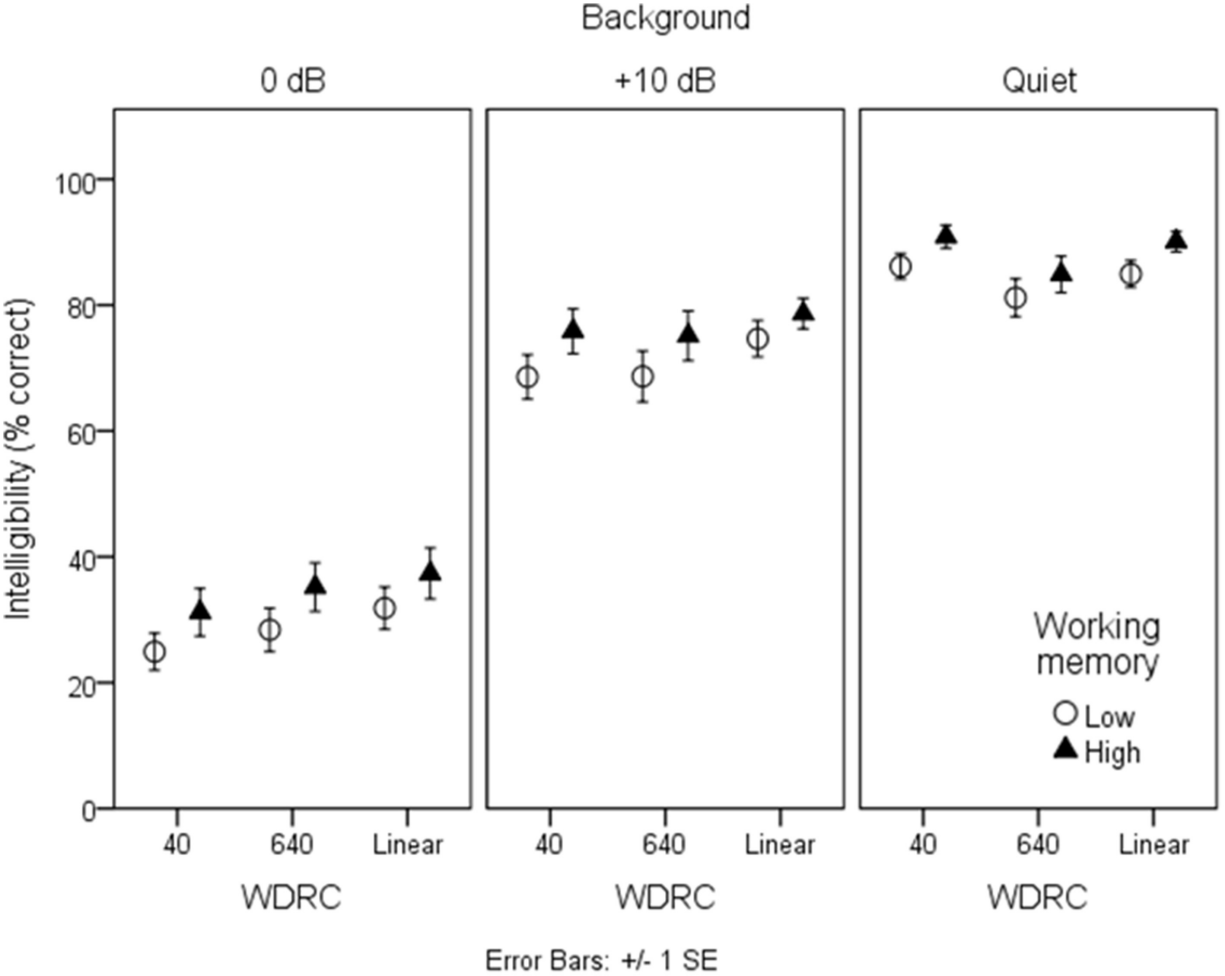
**Mean intelligibility for low- and high-working memory groups by WDRC condition**. Error bars show ± one standard error about the mean.

**Figure 4:**
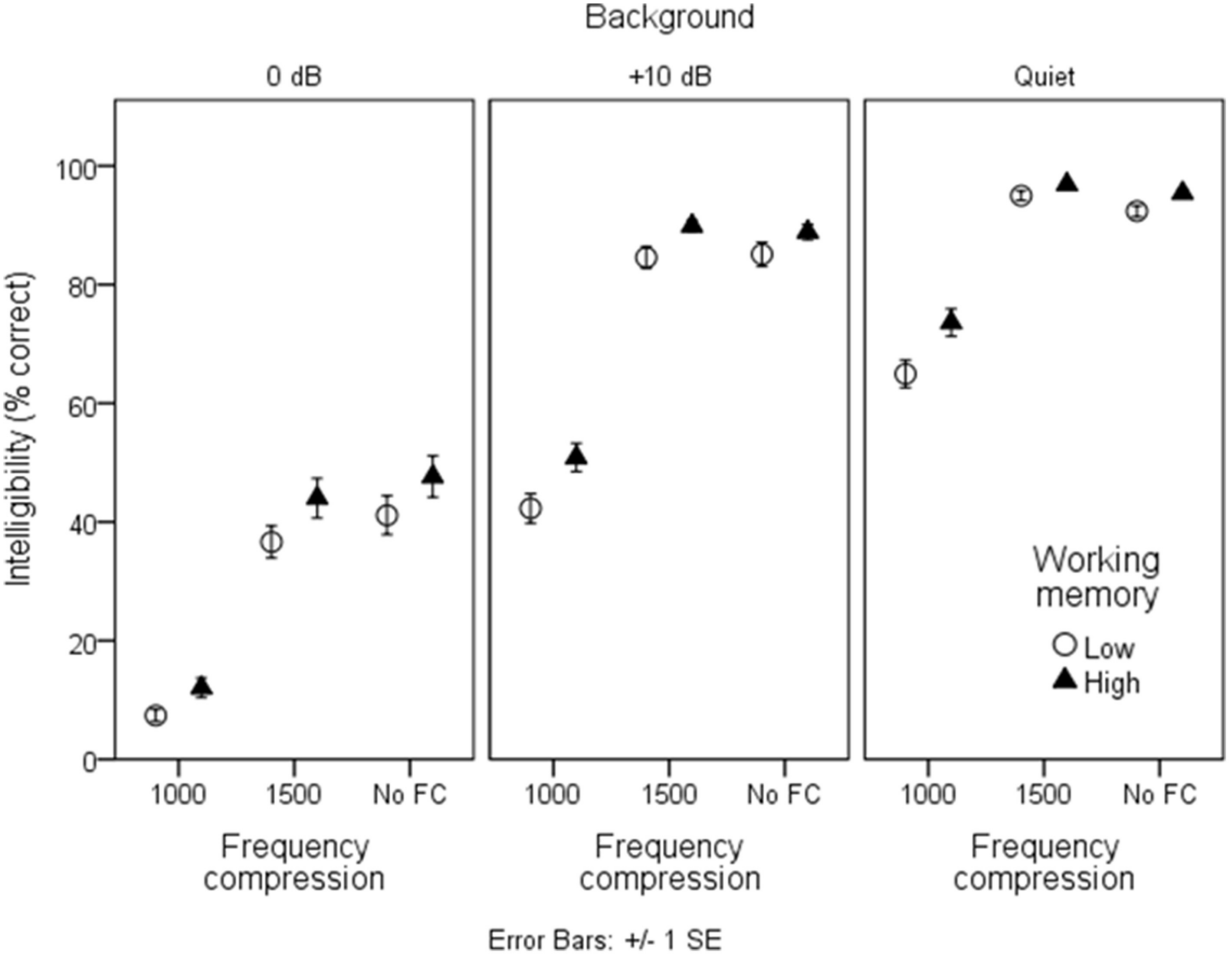
**As in Figure 3, but grouped by frequency compression condition**.

Figures 3, 4 show mean intelligibility scores for each processing condition, grouped by working memory capacity. Recall that signal modification was created by manipulating three aspects of the signal: the amount of background noise; the WDRC release time; and the FC parameters. In Figure 3, data are plotted for the three WDRC conditions (collapsed across FC). In Figure 4, data are plotted for the three FC conditions (collapsed across WDRC). Each panel shows a different signal-to-noise ratio. Several trends are apparent. Scores were lower with more background noise; with more aggressive FC; and with faster WDRC (although the latter difference was quite small and occurred only at the poorest signal-to-noise ratio). With regard to working memory capacity, listeners with higher working memory performed better than their counterparts with low working memory across all conditions.

**Figure 5:**
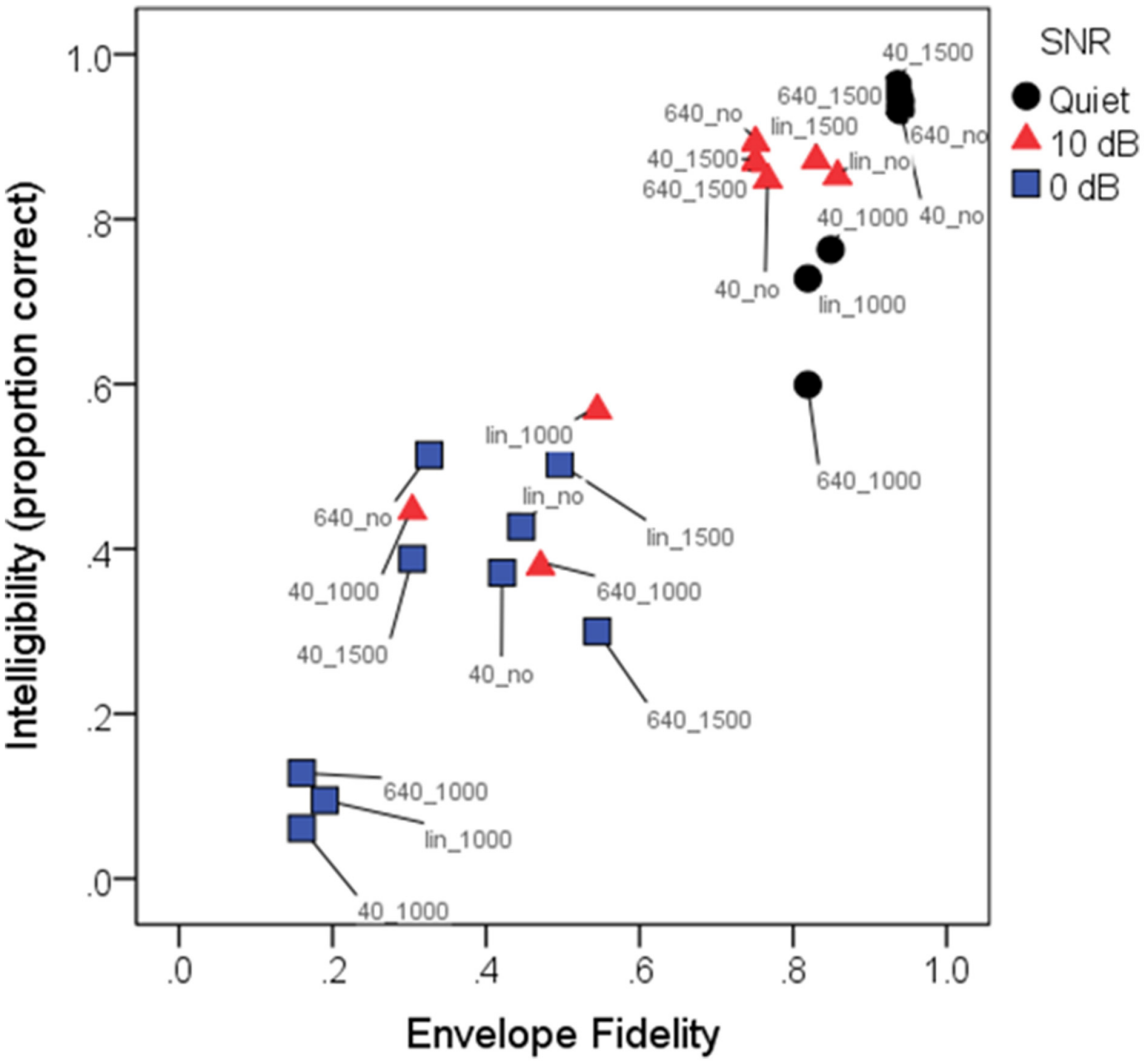
**Mean intelligibility scores (in proportion correct) as a function of envelope fidelity**. For linearity prior to analysis, the envelope distortion metric was subjected to a sigmoidal transformation. Pearson product-moment correlation was 0.93, indicating that the envelope fidelity metric was a good predictor of intelligibility scores. Each condition is indicated by a color-label combination. Symbols indicate the three SNRs: quiet (black circles); 10 dB SNR (red triangles); 0 dB (blue squares). Labels indicate the conditions, where 40_, 640_, and lin_ represent WDRC release time or linear amplification, and _1000, _1500, or _no represent frequency compression cutoff frequency or no FC. As quantified by the envelope fidelity metric, the highest-fidelity condition was linear amplification without frequency compression for speech in quiet, and the lowest-fidelity condition was speech at a signal-to-noise ratio of 0 dB with a compression release time of 40 ms and a frequency compression cutoff of 1000 Hz. Note that due to the close clustering of symbols with high (near-100%) intelligibility, not all symbols and labels are visible in the figure.

The rationale for the various background noise levels and the WDRC and FC processing was to create a range of signal modification, which was expected to underlie intelligibility (and perhaps quality) results. Figure 5 shows average intelligibility scores as a function of the envelope fidelity metric. The envelope fidelity metric was subjected to a sigmoidal transformation to better support the model's assumption of linearity prior to HLM analysis. Each processing combination is indicated by data point labeling, and signal-to-noise ratio is indicated by symbol shape/color. Overall, there was a strong linear relationship between speech intelligibility and the (transformed) fidelity metric (*R*^2^ = 0.88).

#### Model fit and definitions

The multi-level model for this analysis had two levels. The first level represented the individual linear relationship between speech intelligibility and envelope fidelity using estimated intercepts and slope coefficients. Listeners were then classified into groups based on their individual characteristics as described in the analysis section. Those groupings represented the model's second level, where listener characteristics were used to predict variability in the level one coefficients of intercept and slope. If un-centered, the intercept coefficient would have represented speech intelligibility at an envelope fidelity value of zero, where signal modification was very high with minimal between-group differences. Accordingly, we centered the intercept at the mean of the envelope fidelity scale. Centering at the mean of the scale provided a more informative estimation of between group differences.

#### Between-listener variability and descriptive statistics

The average estimated intelligibility for intercept across all listeners and conditions was 63.5% (*SD* = 9%) and the average estimate for slope was 1 (*SD* = 0.08). To get a reference as to the magnitude of between-group differences in intercept and slope, we calculated the predicted 95% range for each coefficient. The predicted range for speech intelligibility intercept was 45.84 to 81.14% and the range for slope was 0.84 to 1.16. Recall that to predict between-listener variability, we explored a hierarchy of conditional models for each cognitive measure (working memory, executive function and processing speed).

Working memory scores (in proportion correct) ranged from 0.19 to 0.59, with a mean score of 0.38. The average processing speed score was 478 ms (range 361 to 606 ms). The average executive function score was 46 ms (range −64 to 204 ms). Correlations between the three cognitive measures (Table 1) were low and were not significant, suggesting that the three measures represented different cognitive domains.

**Table 1:** **Pearson product-moment correlations between cognitive measures**.

	**Processing speed (ms)**	**Executive Function (ms)**	**Working memory (% correct)**
Processing speed (ms)	1.00	−0.07	0.16
Executive function (ms)		1.00	−0.10
Working memory capacity (% correct)			1.00

#### Hierarchical linear model

The HLM model building process included predictors stepwise in an effort to partial out the amount of variability explained as well as the effect size for different listener factors. In each model, the first step included one of the three cognitive measures. In step 2 amount of hearing loss was added, followed by age in the third step.

Table 2 provides a summary of the fixed effects for the working memory model hierarchy. In step 1 the results show that there was a significant positive effect for envelope fidelity on speech intelligibility (*p* < 0.001). However there was no main effect for working memory capacity on intercept or slope. In step 2, when amount of hearing loss (pure-tone average, PTA) was added to the model, there was a significant main effect for working memory capacity (*p* = 0.032) and amount of hearing loss (*p* < 0.001) on intercept but no effect for either factor on slope. In other words, after controlling for amount of hearing loss there was a significant difference in speech intelligibility between the high and low working memory groups when envelope fidelity was at the mean of its scale. In step 3, age was added to the model but did not demonstrate any significant effects.

**Table 2:** **Summary of hierarchical linear model for intelligibility with working memory capacity (WM) as a predictor**.

** Fixed effect**	**Coeff**.	**Std. error**	**T-ratio**	***d.f***	***P*-value**
**STEP 1**						
**For Intercept**						
Intercept	0.604	0.026	23.675	27	<0.001
WM	0.061	0.033	1.860	27	0.073
**For Slope**						
Fidelity index	1.048	0.023	45.665	27	<0.001
WM	−0.070	0.042	−0.649	27	0.110
**STEP 2**						
**For Intercept**						
Intercept	0.603	0.023	26.132	26	<0.001
WM	0.063	0.028	2.269	26	0.032
PTA	−0.003	0.001	−5.935	26	<0.001
**For Slope**						
Fidelity index	1.049	0.024	42.915	26	<0.001
WM	−0.071	0.041	−1.733	26	0.094
PTA	0.002	0.001	1.815	26	0.081
**STEP 3**						
**For Intercept**						
Intercept	0.606	0.021	28.792	25	<0.001
WM	0.056	0.027	2.115	25	0.044
PTA	−0.003	0.0004	−7.001	25	<0.001
Age	−0.001	0.001	−0.881	25	0.387
**For Slope**						
Fidelity index	1.052	0.026	40.404	25	<0.001
WM	−0.076	0.042	−1.774	25	0.088
PTA	0.002	0.001	−1.820	25	0.080
Age	0.000	0.002	−0.325	25	0.748
**STEP 4**						
**For Intercept**						
Intercept	0.737	0.036	20.26	26	<0.001
WM	0.063	0.027	2.269	26	0.032
PTA	−0.003	0.001	−5.935	26	<0.001
**For Slope**						
Fidelity index	1.090	0.066	16.560	25	<0.001
WM	−0.265	0.086	−3.089	25	0.005
PTA	−0.001	0.001	−0.765	25	0.451
WM by PTA	0.005	0.002	2.747	25	0.011

*Amount of hearing loss (PTA) is average of thresholds at 1, 2, 3, and 4 kHz in the test ear*.

The change in the effect of working memory with the addition of amount of hearing loss indicated the presence of an underlying interaction. In step four, we removed age from the model and added a three way interaction (working memory by amount of hearing loss by envelope fidelity). The results of the final model demonstrated significant effects for working memory capacity (*p* = 0.032) and amount of hearing loss (*p* < 0.001) on intercept and a significant effect for working memory (*p* = 0.005) on slope. There was also a significant main effect for the three way interaction on speech intelligibility (*p* = 0.011).

Tables 3, 4 provide the model outcomes when executive function and processing speed were considered the primary cognitive predictor. Neither of these factors was significant predictors of speech intelligibility, either independently or when controlling for amount of hearing loss and age.

**Table 3:** **Summary of hierarchical linear model for intelligibility with executive function (EF) as a predictor**.

** Fixed effect**	**Coeff**.	**Std. error**	**T-ratio**	***d.f***	***P*-value**
**STEP 1**						
**For Intercept**						
Intercept	0.644	0.029	22.403	26	<0.001
EF	−0.026	0.035	−0.758	26	0.455
**For Slope**						
Fidelity index	1.025	0.033	31.014	26	<0.001
EF	−0.013	0.045	−0.286	26	0.777
**STEP 2**						
**For Intercept**						
Intercept	0.769	0.043	17.923	25	<0.001
EF	−0.018	0.030	−0.615	25	0.544
PTA	−0.003	0.001	−4.310	25	<0.001
**For Slope**						
Fidelity index	0.943	0.070	13.495	25	<0.001
EF	−0.018	0.042	−0.423	25	0.676
PTA	0.002	0.001	1.638	25	0.114
**STEP 3**						
**For Intercept**						
Intercept	0.951	0.098	9.708	24	<0.001
EF	−0.014	0.030	−0.461	24	0.649
PTA	−0.003	0.001	−6.048	24	<0.001
Age	0.003	0.002	−1.621	24	0.118
**For Slope**						
Fidelity index	0.879	0.193	4.556	24	<0.001
EF	−0.020	0.045	−0.439	24	0.664
PTA	0.002	0.001	1.687	24	0.104
Age	0.001	0.003	0.336	24	0.740

*Amount of hearing loss (PTA) is average of thresholds at 1, 2, 3, and 4 kHz in the test ear*.

**Table 4:** **Summary of hierarchical linear model for intelligibility with processing speed (PS) as a predictor**.

** Fixed effect**	**Coeff**.	**Std. error**	**T-ratio**	***d.f***	***P*-value**
**STEP 1**						
**For Intercept**						
Intercept	0.626	0.020	30.675	26	<0.001
PS	0.008	0.034	0.246	26	0.808
**For Slope**						
Fidelity index	1.029	0.033	30.624	26	<0.001
PS	−0.022	0.044	−0.503	26	0.619
**STEP 2**						
**For Intercept**						
Intercept	0.763	0.038	20.001	25	<0.001
PS	−0.003	0.029	−0.110	25	0.914
PTA	−0.003	0.001	−3.948	25	0.001
**For Slope**						
Fidelity index	0.946	0.072	13.105	25	<0.001
PS	−0.015	0.043	−0.353	25	0.726
PTA	0.002	0.001	1.523	25	0.140
**STEP 3**						
**For Intercept**						
Intercept	0.951	0.10	8.671	24	<0.001
PS	−0.001	0.027	−0.040	24	0.969
PTA	−0.003	0.001	−5.637	24	<0.001
Age	−0.003	0.001	−1.755	24	0.092
**For Slope**						
Fidelity index	0.886	0.190	4.665	24	<0.001
PS	−0.016	0.043	−0.371	24	0.713
PTA	0.002	0.001	1.548	24	0.134
Age	0.001	0.002	0.338	24	0.738

*Amount of hearing loss (PTA) is average of thresholds at 1, 2, 3, and 4 kHz in the test ear*.

#### Effect sizes and prototypical plots

The working memory model represented in step 4 of Table 2 explained 33% of variability in intercept and 21% of variability in slope. When controlling for amount of hearing loss, listeners in the higher working memory group had an estimated gain of 6.3% in intelligibility at the mean envelope fidelity. As expected, speech intelligibility scores decreased as envelope fidelity decreased. However, after controlling for amount of loss and the hearing loss-by-working memory interaction, listeners' scores in the high working memory group decreased at a slower rate (8.2% per fidelity unit) when compared to listeners in the low working memory group (10% per fidelity unit). Finally, the interaction demonstrated that as envelope fidelity decreased, listeners with milder hearing loss and high working memory capacity tended to have higher intelligibility scores compared to listeners with milder hearing loss and low working memory capacity. As hearing loss increased, the relationship between working memory and speech intelligibility diminished.

**Figure 6:**
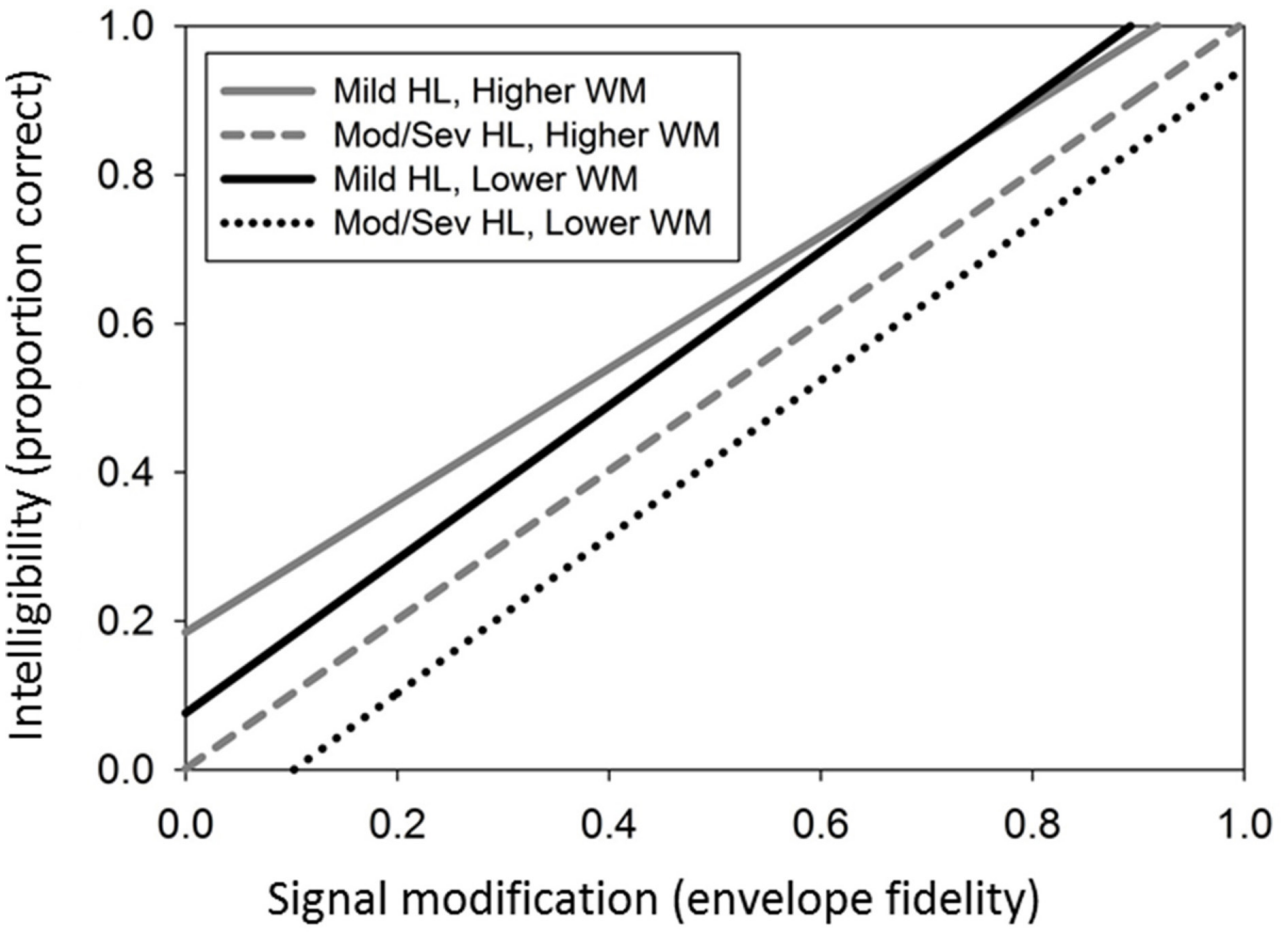
**Final model for intelligibility, showing four different fitted trajectories of intelligibility as a function of envelope fidelity for hearing loss (HL) and working memory (WM)**.

To illustrate the simultaneous effects of all the predictors in the final model for RST, we created a model plot with prototypical listener characteristics. Figure 6 illustrates the model for intelligibility in step 4 and provides four different fitted trajectories of intelligibility as a function of envelope fidelity. The fitted trajectories represented two subsets of listeners within the High and Low working memory groups. In the first subset hearing loss was modeled at the 25th percentile (28 dB HL pure-tone average) and for the second subset hearing loss was modeled at the 75th percentile (49 dB HL pure-tone average).

### Speech quality

**Figure 7:**
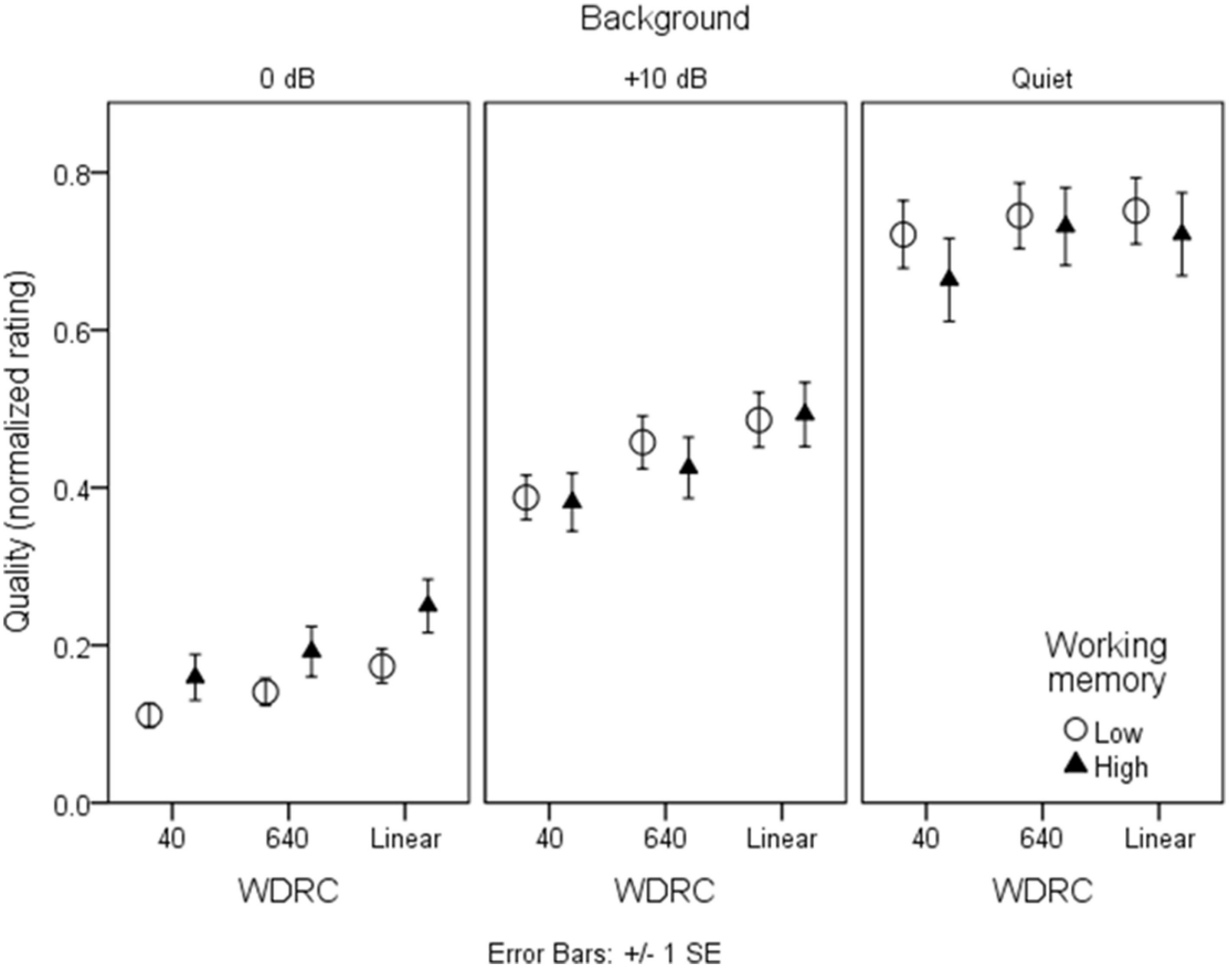
**As in Figure 3, but for quality ratings grouped by WDRC condition**.

**Figure 8:**
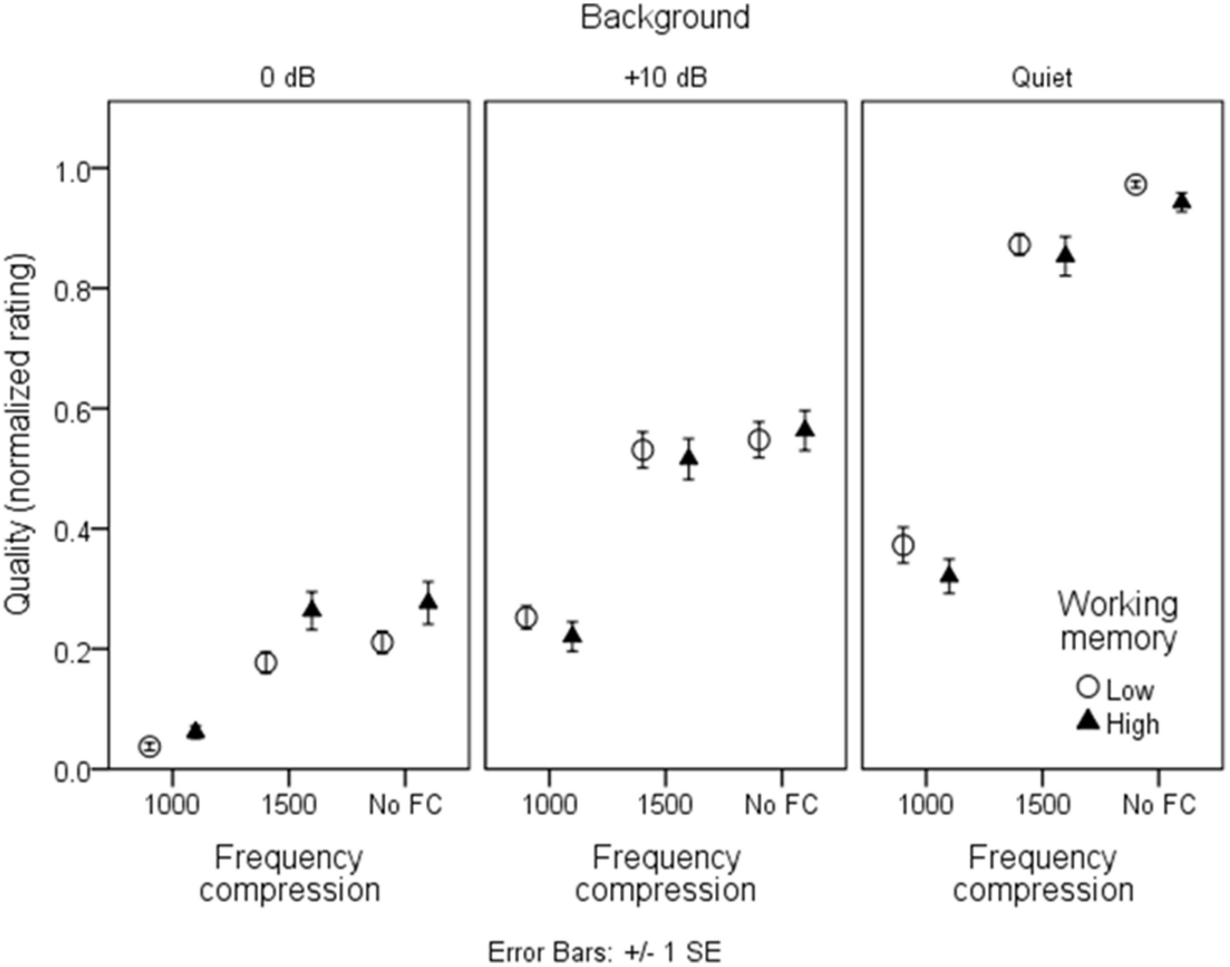
**As in Figure 3, but for quality ratings grouped by frequency compression condition**.

**Figure 9:**
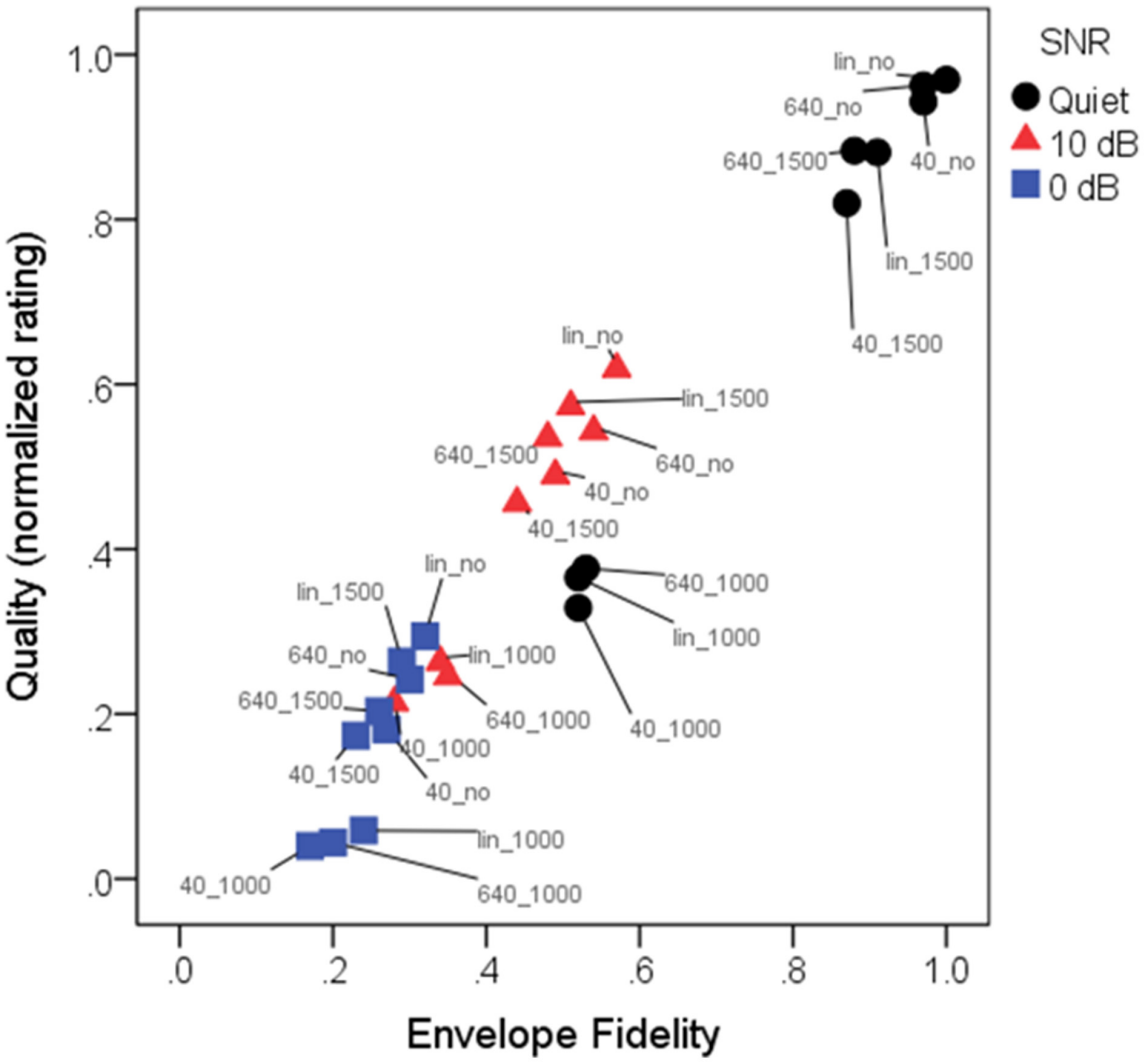
**Mean normalized quality ratings as a function of envelope fidelity**. Pearson product-moment correlation was 0.97, indicating that the envelope fidelity measure was a good predictor of quality ratings. Each condition is indicated by a color-label combination. Symbols indicate the three SNRs: quiet (black circles); 10 dB SNR (red triangles); 0 dB (blue squares). Labels indicate the conditions, where 40_, 640_, and lin_ represent WDRC release time or linear amplification, and _1000, _1500, or _no represent frequency compression cutoff frequency or no FC. As quantified by the envelope fidelity metric, the highest-fidelity condition was linear amplification without frequency compression for speech in quiet, and the lowest-fidelity condition was speech at a signal-to-noise ratio of 0 dB with a compression release time of 40 ms and a frequency compression cutoff of 1000 Hz.

Figures 7, 8 show mean quality ratings for each processing condition. For consistency with the intelligibility figures, listeners are grouped by working memory. In Figure 7, data are plotted for the three WDRC conditions (collapsed across FC). In Figure 8, data are plotted for the three FC conditions (collapsed across WDRC). Each panel shows a different signal-to-noise ratio. In contrast to the intelligibility data (Figures 3, 4), there was no suggestion that working memory capacity influenced quality ratings in a consistent way. However, we anticipated that quality ratings would depend to a large extent on signal modification. Figure 9 shows average quality ratings as a function of the envelope fidelity metric. Each processing combination is indicated by data point labeling, and signal-to-noise ratio is indicated by symbol shape/color. There was a strong linear relationship between speech quality and the fidelity metric (*R*^2^ = 0.88).

#### Between-group variability

The average estimate for quality intercept across all listeners and conditions was 0.44 (*SD* = 0.08) and the average estimate for slope was 1.1 (*SD* = 0.14). The predicted 95% range for quality intercept was 0.28 to 0.60 and the range for slope was 0.83 to 1.37.

#### Hierarchical linear model

Similar to the speech intelligibility analysis, we also included three HLM models for quality in order to identify the independent effect for each cognitive measure. The model building process included predictors stepwise where the first step included one of the three cognitive measures independently. The next step added PTA as a covariate and the third step added age also as a covariate to the model.

Tables 5–7 summarize the parameter coefficients for each HLM model and sub-models provide for quality. The first level model demonstrated that there was a statistically significant effect for envelope fidelity (*p* < 0.001) on quality ratings. For the working memory model, we found no significant effects for working memory group, amount of hearing loss, or age. Similarly, there were no significant effects for processing speed group, amount of hearing loss, or age in the processing speed model (Table 7). The executive function model did reveal a small significant effect for executive function group and age on intercept.

**Table 5:** **Summary of hierarchical linear model for quality with working memory capacity (WM) as a predictor**.

** Fixed effect**	**Coeff**.	**Std. error**	**T-ratio**	***d.f***	***P*-value**
**STEP 1**						
**For Intercept**						
Intercept	0.446	0.019	22.759	27	<0.001
WM	−0.005	0.032	−0.144	27	0.887
**For Slope**						
Fidelity index	1.127	0.032	29.444	27	<0.001
WM	−0.056	0.065	−0.868	27	0.393
**STEP 2**						
**For Intercept**						
Intercept	0.461	0.043	10.817	26	<0.001
WM	−0.004	0.031	−0.129	26	0.899
PTA	−0.0007	0.001	−0.642	26	0.526
**For Slope**						
Fidelity index	1.014	0.107	9.480	26	<0.001
WM	−0.058	0.063	−0.918	26	0.367
PTA	0.003	0.003	1.154	26	0.259
**STEP 3**						
**For Intercept**						
Intercept	0.249	0.111	2.246	25	0.034
WM	0.011	0.031	0.360	25	0.721
PTA	−0.001	0.001	−0.572	25	0.572
Age	0.003	0.002	1.886	25	0.071
**For Slope**						
Fidelity index	1.164	0.295	3.944	25	0.001
WM	−0.069	0.063	−1.082	25	0.290
PTA	0.003	0.003	1.135	25	0.268
Age	−0.002	0.003	−0.572	25	0.572

*Amount of hearing loss (PTA) is average of thresholds at 1, 2, 3, and 4 kHz in the test ear*.

**Table 6:** **Summary of hierarchical linear model for quality with executive function (EF) as a predictor**.

** Fixed effect**	**Coeff**.	**Std. error**	**T-ratio**	***d.f***	***P*-value**
**STEP 1**						
**For Intercept**						
Intercept	0.473	0.022	21.186	26	<0.001
EF	−0.058	0.031	−1.867	26	0.073
**For Slope**						
Fidelity index	1.081	0.039	27.858	26	<0.001
EF	0.028	0.067	0.415	26	0.681
**STEP 2**						
**For Intercept**						
Intercept	0.491	0.050	9.765	25	<0.001
EF	−0.057	0.030	−1.874	25	0.072
PTA	−0.000	0.001	−0.483	25	0.633
**For Slope**						
Fidelity index	0.975	0.113	8.637	25	<0.001
EF	0.021	0.067	0.313	25	0.757
PTA	0.003	0.003	1.109	25	0.279
**STEP 3**						
**For Intercept**						
Intercept	0.273	0.087	3.145	24	0.005
EF	−0.062	0.029	−2.160	24	0.040
PTA	−0.0004	0.001	−0.430	24	0.670
Age	0.003	0.001	2.388	24	0.025
**For Slope**						
Fidelity index	1.081	0.280	3.867	24	0.001
EF	0.024	0.069	0.344	24	0.733
PTA	0.003	0.003	1.076	24	0.293
Age	−0.001	0.003	−0.433	24	0.669

*Amount of hearing loss (PTA) is average of thresholds at 1, 2, 3, and 4 kHz in the test ear*.

**Table 7:** **Summary of hierarchical linear model for quality with processing speed (PS) as a predictor**.

** Fixed effect**	**Coeff**.	**Std. error**	**T-ratio**	***d.f***	***P*-value**
**STEP 1**						
**For Intercept**						
Intercept	0.412	0.020	20.783	26	<0.001
PS	0.059	0.031	1.905	26	0.067
**For Slope**						
Fidelity index	1.119	0.038	29.827	26	<0.001
PS	−0.047	0.068	−0.629	26	0.495
**STEP 2**						
**For Intercept**						
Intercept	0.428	0.040	10.786		<0.001
PS	0.057	0.031	1.872		0.072
PTA	−0.000	0.001	−0.432		0.669
**For Slope**						
Fidelity index	1.009	0.113	8.927	25	<0.001
PS	−0.038	0.067	−0.565	25	0.575
PTA	0.003	0.002	1.103	25	0.281
**STEP 3**						
**For Intercept**						
Intercept	0.239	0.095	2.525	24	0.019
PS	0.055	0.030	1.863	24	0.074
PTA	−0.000	0.001	−0.373	24	0.712
Age	0.003	0.001	1.968	24	0.060
**For Slope**						
Fidelity index	1.102	0.274	4.018	24	0.001
PS	−0.037	0.067	−0.544	24	0.591
PTA	0.003	0.002	1.079	24	0.292
Age	−0.001	0.003	−0.383	24	0.705

*Amount of hearing loss (PTA) is average of thresholds at 1, 2, 3, and 4 kHz in the test ear*.

## Discussion

Our first question concerned speech intelligibility (and quality) across adverse listening conditions. We considered “adverse” quite broadly to mean addition of background noise and/or modifications of the acoustic signal (here, by WDRC and FC). An envelope fidelity metric was used to quantify those modifications. Speech intelligibility and quality were well predicted by the envelope fidelity metric.

Next, we explored the role of listener factors on speech intelligibility (and quality) performance under adverse listening conditions. The patient factors that were considered were amount of hearing loss, age, working memory capacity, executive function and processing speed. The focus of the study was working memory capacity, which had already been shown to be related to hearing aid processing parameters when a single type of processing was applied. A recent model of working memory (Rönnberg et al., 2013) suggests that when signal modification impedes a rapid match of acoustic information to stored representations, working memory will be engaged. In that situation, listeners with low working memory capacity may be at a disadvantage. The present results were in good agreement with that expectation. Specifically, listeners with low working memory capacity (as quantified by a RST) performed more poorly for a given amount of signal modification (as quantified by the envelope fidelity metric) compared to individuals with high working memory capacity. That difference occurred despite having similar amount of hearing loss and age. Our results were consistent with the literature in showing the effect of working memory capacity on speech recognition. They also add to the literature regarding single-feature manipulations, from fast-acting WDRC (e.g., Gatehouse et al., 2006) and FC (e.g., Arehart et al., 2013a).

We also hypothesized that listeners with low working memory capacity would be disproportionately affected by high amounts of signal modification. Results of HLM modeling of intelligibility slope supported this hypothesis, although the effect also depended on the amount of hearing loss. In a general sense, the statistical result highlights the accumulating factors, with the poorest recognition of distorted signals by listeners with more hearing loss and with low working memory capacity. Our data reinforce results of Neher (2014), in which substantial variance in intelligibility was explained by amount of hearing loss and by working memory capacity.

Speech quality ratings were related to signal fidelity, but not to working memory capacity. There was a small effect of executive function on quality. Although our measure relied on rated speech quality rather than preference, and although we used the addition of background noise rather than noise reduction, this is generally consistent with Neher's (2014) finding that the preferred noise reduction setting depended on executive function (assuming that sound quality is a criterion for preference).

From a diagnostic standpoint, it is of interest to know whether one cognitive factor (here, working memory capacity, executive function, or processing speed) is a stronger predictor of intelligibility in adverse listening conditions. We hypothesized that individuals with lower executive function and/or slower processing speed might be more affected when adverse listening environments are created by signal modification. However, processing speed and executive function did not explain a significant proportion of the variance in speech intelligibility. Neher (2014) also examined the influence of executive function (specifically, the ability to maintain focus on relevant information) on speech modified by hearing-aid (noise reduction) processing. Consistent with our results, Neher reported that executive function accounted for a very small portion (3%) of the variance in a speech intelligibility task, and reported weak correlations among working memory (via a RST) and executive function. Overall, these findings suggest minimal influence of processing speed and executive function on speech intelligibility, but some qualifications are worth noting. First, in the present data and in Neher (2014), working memory capacity was measured using a linguistic paradigm, while processing speed and executive function were measured using non-linguistic paradigms. It is likely that these non-linguistic paradigms did not capture the variability in top-down linguistic processing of sentence stimuli, which is a critical ability exploited by older listeners to compensate for distorted speech signals in challenging listening situations (Pichora-Fuller, 2008). Second, the speech intelligibility tasks used in both studies were directed speech tasks, in the sense that the listener's attention was pre-focused on the speech-in-noise signal. That presentation differs from many everyday situations in which the listener must direct attention among different talkers, potentially engaging executive function to a greater extent. It is possible that other measures of executive function and/or other speech scenarios might produce different results.

The present data (following the recent paper by Neher, 2014) add a multi-dimensional understanding of the relationship between working memory capacity and the characteristics of the speech signal, demonstrating that the relationship persists when signal modification is introduced via a combination of signal processing approaches. From a research perspective, these data are important as we refine our understanding of the role of working memory in adverse situations. From a translational perspective, these findings provide support for the idea that individuals with low working memory capacity might achieve better intelligibility with signal processing that maintains the fidelity of the signal envelope. However, more study is needed to explore the boundaries of the effect with regard to speech materials, noise type, and other aspects of listening, before such recommendations can be implemented in clinical practice. In particular, other aspects of hearing aid processing may produce different results. For example, the goal of noise suppression is to restore changes to the speech envelope caused by additive noise. Therefore, the cumulative effects of hearing aid signal processing that combines noise suppression with fast-acting WDRC and FC may differ from the results reported here. Finally, in the present study, the signal processing parameters were selected relative to our experimental goals, rather than customized for individual listeners. In future work, it will be important to consider both the effects of combined signal processing and customization of that processing to listener needs.

### Conflict of interest statement

The authors declare that the research was conducted in the absence of any commercial or financial relationships that could be construed as a potential conflict of interest.

## Footnotes

^1^International Telecommunication Union ITU-R: BS.1284-1, “General Methods for the Subjective Assessment of Sound Quality” (2003).

## References

[B1] Akeroyd M. A. (2008). Are individual differences in speech reception related to individual differences in cognitive ability? A survey of twenty experimental studies with normal and hearing-impaired adults. Int. J. Audiol. 47 (Suppl. 2), S53–S71. 10.1080/14992020802301142 19012113

[B2] Alexander J. M. Kopun J. G. Stelmachowicz P. G. (2014). Effects of frequency compression and frequency transposition on fricative and affricate perception in listeners with normal hearing and mild to moderate hearing loss. Ear Hear. 35, 519–532. 10.1097/AUD.0000000000000040 24699702 PMC4141891

[B3] Anderson M. C. Arehart K. H. Kates J. M. (2009). The acoustic and perceptual effects of series and parallel processing. EURASIP J. Adv. Signal Process. 2009:619805. 10.1155/2009/61980510686183

[B4] Anderson S. Parbery-Clark A. White-Schwoch T. Kraus N. (2012). Aging affects neural precision of speech encoding. J. Neurosci. 32, 14156–14164. 10.1523/JNEUROSCI.2176-12.2012 23055485 PMC3488287

[B5] Anderson S. White-Schwoch T. Parbery-Clark A. Kraus N. (2013). Reversal of age-related neural timing delays with training. Proc. Natl. Acad. Sci. U.S.A. 110, 4357–4362. 10.1073/pnas.1213555110 23401541 PMC3600492

[B6] ANSI (2009). American National Standards Institute Specification of Hearing Aid Characteristics. New York, NY: ANSI.

[B7] Arehart K. H. Kates J. M. Anderson M. C. (2010). Effects of noise, nonlinear processing, and linear filtering on perceived speech quality. Ear Hear. 31, 420–436. 10.1097/AUD.0b013e3181d3d4f3 20440116

[B8] Arehart K. H. Kates J. M. Anderson M. C. Harvey L. O. Jr (2007). Effects of noise and distortion on speech quality judgments in normal-hearing and hearing-impaired listeners. J. Acoust. Soc. Am. 122, 1150–1164. 10.1121/1.2754061 17672661

[B9] Arehart K. H. Souza P. Baca R. Kates J. M. (2013a). Working memory, age, and hearing loss: susceptibility to hearing aid distortion. Ear Hear. 34, 251–260. 10.1097/AUD.0b013e318271aa5e 23291963 PMC3636195

[B10] Arehart K. H. Souza P. Lunner T. Syskind Pedersen M. Kates J. M. (2013b). Relationship between distortion and working memory for digital noise-reduction processing in hearing aids, in Presented at the Acoustical Society of America Convention (Montreal, QC). 10.1121/1.4805834

[B10a] Baddeley A. (1992). Working memory. Science 255, 556–559. 10.1126/science.1736359 1736359

[B11] Baddeley A. (2000). The episodic buffer: a new component of working memory? Trends Cogn. Sci. 4, 417–423. 10.1016/S1364-6613(00)01538-2 11058819

[B12] Besser J. Koelewijn T. Zekveld A. A. Kramer S. E. Festen J. M. (2013). How linguistic closure and verbal working memory relate to speech recognition in noise–a review. Trends Amplif. 17, 75–93. 10.1177/1084713813495459 23945955 PMC4070613

[B13] Byrne D. Dillon H. Ching T. Katsch R. Keidser G. (2001). NAL-NL1 procedure for fitting nonlinear hearing aids: characteristics and comparisons with other procedures. J. Am. Acad. Audiol. 12, 37–51. 11214977

[B14] Chung K. (2007). Effective compression and noise reduction configurations for hearing protectors. J. Acoust. Soc. Am. 121, 1090–1101. 10.1121/1.2409859 17348531

[B15] Cox R. M. Alexander G. C. Gilmore C. (1987). Development of the Connected Speech Test (CST). Ear Hear. 8, 119S–126S. 10.1097/00003446-198710001-00010 3678650

[B16] Daneman M. Carpenter P. A. (1980). Individual differences in working memory and reading. J. Verbal Learn. Verbal Behav. 19, 450–466 10.1016/S0022-5371(80)90312-6

[B17] Davies-Venn E. Souza P. (2014). The role of spectral resolution, working memory, and audibility in explaining variance in susceptibility to temporal envelope distortion. J. Am. Acad. Audiol. 25, 592–604. 10.3766/jaaa.25.6.9 25313549 PMC4412362

[B18] Dupuis K. Marchuk V. Pichora-Fuller M. K. Chasteen A. L. Singh G. Smith S. L. (2013). Sensory loss and performance on the Montreal Cognitive Assessment, in Presented at the Aging and Speech Communication Conference (Bloomington, IN).

[B19] Ellis R. J. Munro K. J. (2015). Benefit from, and acclimitization to, frequency compression hearing aids in experienced adult hearing-aid users. Int. J. Audiol. 54, 37–47. 10.3109/14992027.2014.948217 25470620

[B20] Eriksen B. A. Ericksen C. W. (1974). Effects of noise letters upon the identification of a target letter in a nonsearch task. Percept. Psychophys. 16, 143–149 10.3758/BF03203267

[B21] Foo C. Rudner M. Rönnberg J. Lunner T. (2007). Recognition of speech in noise with new hearing instrument compression release settings requires explicit cognitive storage and processing capacity. J. Am. Acad. Audiol. 18, 618–631. 10.3766/jaaa.18.7.8 18236648

[B22] Franck B. A. M. van Kreveld-Box C. S. G. M. Dreschler W. A. (1999). Evaluation of spectral enhancement in hearing aids, combined with phonemic compression. J. Acoust. Soc. Am. 106, 1452–1464. 10.1121/1.428055 10489703

[B23] Fry A. F. Hale S. (1996). Processing speed, working memory, and fluid intelligence: evidence for a developmental cascade. Psychol. Sci. 7, 237–241. 10.1111/j.1467-9280.1996.tb00366.x11035218

[B24] Gabrielsson A. Schenkman B. N. Hagerman B. (1988). The effects of different frequency responses on sound quality judgments and speech intelligibility. J. Speech Hear. Res. 31, 166–177. 10.1044/jshr.3102.166 3398490

[B25] Gatehouse S. Naylor G. Elberling C. (2006). Linear and nonlinear hearing aid fittings–2. Patterns of candidature. Int. J. Audiol. 45, 153–171. 10.1080/14992020500429484 16579491

[B26] George E. L. Zekveld A. A. Kramer S. E. Goverts S. T. Festen J. M. (2007). Auditory and nonauditory factors affecting speech recognition. J. Acoust. Soc. Am. 121, 2362–2375. 10.1121/1.2642072 17471748

[B27] Hodgson M. Steininger G. Razavi Z. (2007). Measurement and prediction of speech and noise levels and the Lombard effect in eating establishments. J. Acoust. Soc. Am. 121, 2023–2033. 10.1121/1.2535571 17471719

[B28] Humes L. Floyd S. S. (2005). Measures of working memory, sequence learning, and speech recognition in the elderly. J. Speech Lang. Hear. Res. 48, 224–235. 10.1044/1092-4388(2005/016) 15938066

[B29] Jenstad L. M. Souza P. E. (2005). Quantifying the effect of compression hearing aid release time on speech acoustics and intelligibility. J. Speech Lang. Hear. Res. 48, 651–667. 10.1044/1092-4388(2005/045) 16197279

[B30] Kates J. M. (2008). Digital Hearing Aids. San Diego, CA: Plural Publishing.

[B31] Kates J. M. Arehart K. H. (2014). The Hearing Aid Speech Perception Index (HASPI). Speech Commun. 65, 75–93 10.1016/j.specom.2014.06.002

[B32] Kates J. M. Arehart K. H. Souza P. (2013). Integrating cognitive and peripheral factors in predicting hearing-aid processing benefit. J. Acoust. Soc. Am. 134, 4458–4469. 10.1121/1.4824700 25669257 PMC3874061

[B33] Lunner T. Sundewall-Thoren E. (2007). Interactions between cognition, compression, and listening conditions: effects on speech-in-noise performance in a two-channel hearing aid. J. Am. Acad. Audiol. 18, 604–617. 10.3766/jaaa.18.7.7 18236647

[B34] McAulay R. J. Quatieri T. F. (1986). Speech analysis/synthesis based on a sinusoidal representation. IEEE Trans. Acoust. Speech Signal Process. 34, 744–754 10.1109/TASSP.1986.1164910

[B35] McDermott H. J. (2011). A technical comparison of digital frequency-lowering algorithms available in two current hearing aids. PLoS ONE 6:e22358. 10.1371/journal.pone.0022358 21789254 PMC3137629

[B36] Miyake A. Emerson M. J. Friedman N. P. (2000). Assessment of executive functions in clinical settings: problems and recommendations. Semin. Speech Lang. 21, 169–183. 10.1055/s-2000-7563 10879548

[B37] Moore B. C. (2004). Dead regions in the cochlea: conceptual foundations, diagnosis and clinical applications. Ear Hear. 25, 98–116. 10.1097/01.AUD.0000120359.49711.D7 15064655

[B38] Nasreddine Z. S. Phillips N. A. Bedirian V. Charbonneau S. Whitehead V. Collin I.. (2005). The Montreal Cognitive Assessment, MoCA: a brief screening tool for mild cognitive impairment. J. Am. Geriatr. Soc. 53, 695–699. 10.1111/j.1532-5415.2005.53221.x 15817019

[B39] Neher T. (2014). Relating hearing loss and executive functions to hearing aid users' preference for, and speech recognition with, different combinations of binaural noise reduction and microphone directionality. Front. Neurosci. 8:391. 10.3389/fnins.2014.00391 25538547 PMC4255521

[B40] Neher T. Grimm G. Hohmann V. (2014). Perceptual consequences of different signal changes due to binaural noise reduction: do hearing loss and working memory capacity play a role? Ear Hear. 35, e213–e227. 10.1097/AUD.0000000000000054 25010636

[B41] Neher T. Grimm G. Hohmann V. Kollmeier B. (2013). Do hearing loss and cognitive function modulate benefit from different binaural noise-reduction settings? Ear Hear. 35, e52–e62. 10.1097/AUD.0000000000000003 24351610

[B43] Ohlenforst B. Souza P. MacDonald E. (2014). Interaction of working memory, compressor speed and background noise characteristics, in Presented at the American Auditory Society Conference, (Scottsdale, AZ).

[B44] Olsen W. O. (1998). Average speech levels spectra in various speaking/listening conditions: a summary of the Pearson, Bennett and Fidell (1977 report. Am. J. Audiol. 7, 21–25 10.1044/1059-0889(1998/012) 26649514

[B45] Pichora-Fuller M. K. (2008). Use of supportive context by younger and older adult listeners: balancing bottom-up and top-down information processing. Int. J. Audiol. 47, S72–S82. 10.1080/14992020802307404 19012114

[B46] Pichora-Fuller M. K. Souza P. (2003). Effects of aging on auditory processing of speech. Int. J. Audiol. 42, 2S11–2S16. 10.3109/14992020309074638 12918623

[B47] Raudenbush S. W. Bryk A. S. (2002). Hierarchical Linear Models: Applications and Data Analysis Methods, 2nd Edn. Thousand Oaks, CA: Sage Publishing.

[B48] Rönnberg J. Arlinger S. Lyxell B. Kinnefors C. (1989). Visual evoked potentials: relation to adult speechreading and cognitive function. J. Speech Hear. Res. 32, 725–735. 10.1044/jshr.3204.725 2601304

[B49] Rönnberg J. Lunner T. Zekveld A. Sorqvist P. Danielsson H. Lyxell B.. (2013). The Ease of Language Understanding (ELU) model: theoretical, empirical, and clinical advances. Front. Syst. Neurosci. 7:31. 10.3389/fnsys.2013.00031 23874273 PMC3710434

[B50] Rosenthal S. (1969). IEEE: recommended practices for speech quality measurements. IEEE Trans. Audio Electroacoust. 17, 227–246.

[B51] Rosetti H. C. Lacritz L. H. Cullum C. M. Weiner M. F. (2011). Normative data for the Montreal Cognitive Assessment (MoCA) in a population-based sample. Neurology 77, 1272–1275. 10.1212/WNL.0b013e318230208a 21917776

[B52] Salthouse T. A. (1991). Mediation of adult age difference in cognition by reductions in working memory and speed of processing. Psychol. Sci. 2, 179–183 10.1111/j.1467-9280.1991.tb00127.x

[B53] Salthouse T. A. (2000). Aging and measures of processing speed. Biol. Psychol. 54, 35–54. 10.1016/S0301-0511(00)00052-1 11035219

[B54] Singer J. D. Willett J. B. (2003). Applied Longitudinal Data Analysis: Modeling Change and Event Occurrence. New York, NY: Oxford University Press 10.1093/acprof:oso/9780195152968.001.0001

[B55] Smeda K. Wolters F. Rung M. (2015). Estimation of signal-to-noise ratios in realistic sound scenarios. J. Am. Acad. Audiol. 26, 183–196. 10.3766/jaaa.26.2.7 25690777

[B56] Souza P. Arehart K. H. Kates J. M. Croghan N. B. Gehani N. (2013). Exploring the limits of frequency lowering. J. Speech Lang. Hear. Res. 56, 1349–1363. 10.1044/1092-4388(2013/12-0151) 23785188 PMC3796181

[B57] Souza P. Sirow L. (2014). Relating working memory to compression parameters in clinically fit hearing aids. Am. J. Audiol. 23, 394–401. 10.1044/2014_AJA-14-0006 25123548 PMC4332516

[B58] Zahorian S. A. Rothenberg M. (1981). Principal-components analysis for low-redundancy encoding of speech spectra. J. Acoust. Soc. Am. 69, 832 10.1121/1.385539

